# Complete Genome Sequence of West Nile Virus (WNV) from the First Human Case of Neuroinvasive WNV Infection in Cyprus

**DOI:** 10.1128/genomeA.01110-17

**Published:** 2017-10-26

**Authors:** Jan Richter, Christina Tryfonos, Aristomenis Tourvas, Dora Floridou, Niki I. Paphitou, Christina Christodoulou

**Affiliations:** a Department of Molecular Virology, Cyprus Institute of Neurology and Genetics, Nicosia, Cyprus; b Intensive Care Clinic, Nicosia General Hospital, Nicosia, Cyprus; c American Medical Center, Nicosia, Cyprus

## Abstract

We report here the complete genome sequence of a West Nile virus (WNV) strain from the first laboratory-confirmed human case of neuroinvasive WNV infection in Cyprus. Phylogenetic analysis showed that this WNV strain grouped clearly into genetic lineage 1, clade 1a, cluster 2.

## GENOME ANNOUNCEMENT

West Nile virus (WNV) is a neurotropic mosquito-borne pathogen belonging to the *Flavivirus* genus within the *Flaviviridae* family. The virus is maintained in an enzootic cycle in birds, which act as amplifying hosts for ornithophilic mosquito vectors belonging to *Culex* spp., but it can be transmitted to other mammals, including humans serving as dead-end hosts (1, 2). WNV is an enveloped, single-stranded positive-sense RNA virus of approximately 11 kb. WNV strains have been classified into at least seven putative genetic lineages; however, only lineages 1 and 2 have been associated with significant outbreaks in humans. Recently, we reported the first human case of neuroinvasive West Nile virus infection in Cyprus in an elderly nonimmunosuppressed patient with a clinical picture of rapidly progressing ascending paralysis mimicking Guillain-Barré syndrome (3). The aim of this study was to establish and characterize the complete genomic sequence of the virus isolate obtained from this patient.

Previously, it was shown that whole-transcriptome amplification techniques, which are based on multiple-displacement amplification, can be successfully used for amplification of RNA virus genomes (4). Because of the limited amount of viral genomic material available, a similar approach was employed here using the Quantitect whole-transcriptome kit (Qiagen) to increase the amount of cDNA necessary for sequencing the complete genome. The successful amplification of the viral RNA was followed by Sanger sequencing of overlapping PCR products. Sequenced amplicons were assembled in BioEdit version 7.0.2, and phylogenetic analyses were conducted in MEGA6 (5). The sequenced genome comprises 10,982 nucleotides with a polyprotein of 3,434 amino acids being encoded within a single open reading frame. Phylogenetic analysis showed that the WNV strain from Cyprus grouped clearly into genetic lineage 1, clade 1a, cluster 2, and had the highest nucleotide identity with a strain isolated recently from a dromedary camel in the United Arab Emirates in 2015 (6, 7). Cluster 2 strains have been isolated from European and African epidemics and are believed to originate from North Africa (8). Comparative genome analysis revealed that the Cyprus strain contains the N-linked glycosylation site (NYST) in the E protein, starting at position 154, which is associated with neuroinvasiveness and increased pathogenicity (9).

The first evidence of WNV in Cyprus stems from a serological and ectoparasite survey of migratory birds in the Eastern Mediterranean between 1966 and 1971 conducted by the U.S. Department of Defense. A WNV was isolated from a barred warbler, (*Sylvia nisoria*) that was later sequenced and found to belong to lineage 2 (10). Since Cyprus lies on the path of several major migratory bird routes, the introduction of WNV on the island is expected given that several mosquito species of the genus *Culex* are abundant in Cyprus (11).

In conclusion, we report the complete genome sequence of a West Nile virus strain obtained from the first human case of neuroinvasive WNV infection in Cyprus. Phylogenetic analysis revealed that the virus belongs to lineage 1a.

### Accession number(s).

The nucleotide sequence of the WNV strain WNV_Cy2016 determined in this study has been deposited at GenBank under accession no. MF797870.

## References

[1] Rossi SL, Ross TM, Evans JD 2010 West Nile virus. Clin Lab Med 30:47–65. doi:10.1016/j.cll.2009.10.006. 20513541 PMC2905782

[2] Kramer LD, Li J, Shi PY 2007 West Nile virus. Lancet Neurol 6:171–181. doi:10.1016/S1474-4422(07)70030-3. 17239804

[3] Paphitou NI, Tourvas A, Floridou D, Richter J, Tryfonos C, Christodoulou C 2017 The first human case of neuroinvasive West Nile virus infection identified in Cyprus. J Infect Publ Health. doi:10.1016/j.jiph.2017.02.003. 28233724

[4] Sujayanont P, Chininmanu K, Tassaneetrithep B, Tangthawornchaikul N, Malasit P, Suriyaphol P 2014 Comparison of phi29-based whole genome amplification and whole transcriptome amplification in dengue virus. J Virol Methods 195:141–147. doi:10.1016/j.jviromet.2013.10.005. 24129073

[5] Tamura K, Stecher G, Peterson D, Filipski A, Kumar S 2013 MEGA6: Molecular Evolutionary Genetics Analysis version 6.0. Mol Biol Evol 30:2725–2729. doi:10.1093/molbev/mst197. 24132122 PMC3840312

[6] Joseph S, Wernery U, Teng JL, Wernery R, Huang Y, Patteril NA, Chan KH, Elizabeth SK, Fan RY, Lau SK, Kinne J, Woo PC 2016 First isolation of West Nile virus from a dromedary camel. Emerg Microbes Infect 5:e53. doi:10.1038/emi.2016.53. 27273223 PMC4932647

[7] Lanciotti RS, Ebel GD, Deubel V, Kerst AJ, Murri S, Meyer R, Bowen M, McKinney N, Morrill WE, Crabtree MB, Kramer LD, Roehrig JT 2002 Complete genome sequences and phylogenetic analysis of West Nile virus strains isolated from the United States, Europe, and the Middle East. Virology 298:96–105. doi:10.1006/viro.2002.1449. 12093177

[8] May FJ, Davis CT, Tesh RB, Barrett ADT 2011 Phylogeography of West Nile virus: from the cradle of evolution in Africa to Eurasia, Australia, and the Americas. J Virol 85:2964–2974. doi:10.1128/JVI.01963-10. 21159871 PMC3067944

[9] Hanna SL, Pierson TC, Sanchez MD, Ahmed AA, Murtadha MM, Doms RW 2005 N-Linked glycosylation of West Nile virus envelope proteins influences particle assembly and infectivity. J Virol 79:13262–13274. doi:10.1128/JVI.79.21.13262-13274.2005. 16227249 PMC1262570

[10] Watson GE, Shope RE, Kaiser MN 1972 An ectoparasite and virus survey of migratory birds in the eastern Mediterranean, p 176–180. *In* Cherepanov IA (ed), Transcontinental connections of migratory birds and their role in the distribution of arboviruses. Nauka, Novosibirsk, Russia.

[11] Violaris M, Vasquez MI, Samanidou A, Wirth MC, Hadjivassilis A 2009 The mosquito fauna of the republic of Cyprus: a revised list. J Am Mosq Control Assoc 25:199–202. doi:10.2987/08-5793.1. 19653503

